# Do you feel like being proactive today? Trait-proactivity moderates affective causes and consequences of proactive behavior

**DOI:** 10.1371/journal.pone.0220172

**Published:** 2019-08-13

**Authors:** Inge Wolsink, Deanne D. Den Hartog, Frank D. Belschak, Suzanne Oosterwijk

**Affiliations:** 1 Department of Leadership and Management, Faculty of Economics and Business, University of Amsterdam, Amsterdam, The Netherlands; 2 Department of Social Psychology, Faculty of Social and Behavioural Sciences, University of Amsterdam, Amsterdam, The Netherlands; University of Lleida, SPAIN

## Abstract

Proactive people take initiative when others do not and persist in improving their environment or themselves. Although scholars assume that how we feel influences how proactive we are, there is no experimental research yet to support this. This experiment therefore tests whether positive and negative affect influence proactive behavior and additionally investigates whether engaging in proactivity also has affective consequences. While current theory proposes that positive affect enhances proactive behavior by stimulating broad-flexible thinking, we argue that negative affect should make people proactive through stimulating systematic-persistent thinking. Furthermore, we propose that proactive behavior increases subsequent positive affect rather than positive affect increasing proactive behavior. Last, we hypothesize that affective causes and consequences of proactive behavior are different for people who are rarely proactive (trait-passive-reactive individuals) and people who are often proactive (trait-proactive individuals). We pre-tested 180 participants on trait-proactivity. In the lab, we manipulated affect (negative/positive/neutral), measured proactive behavior in a team interaction task, and repeatedly measured participants’ affective experiences and physiological activation. Results showed that the link between affect and proactive behavior differed depending on participants’ trait-proactivity. First, positive affect made trait-proactive individuals less proactive, whereas negative affect made passive-reactive individuals more proactive. Second, passive-reactive individuals reported decreased negative affect after engaging in proactivity, whereas proactive individuals reported increased positive affect. These results suggest that proactive behavior can serve an affect regulation purpose, which is different for trait proactive individuals (up regulating positive affect) than for trait passive-reactive individuals (down regulating negative affect). These results are limited to core affect (feeling pleasant or unpleasant) and do not apply to specific emotions (feeling proud or anxious), and they are limited to short term and successful proactive behavior and do not apply to more long term, or unsuccessful proactive behavior.

## Introduction

“The best way to not feel hopeless is to get up and do something. Don’t wait for good things to happen to you. If you go out and make some good things happen, you will fill the world with hope, you will fill yourself with hope.”

Barack Obama

Proactive individuals ‘get up and do something’. They do not merely react to instructions or situational demands, but instead initiate anticipatory action to change processes, tackle problems, and persist in trying to improve things even when they encounter obstacles along the way [1, 2]. Proactive behavior is thus self-starting, challenging, future focused, and persistent in the face of setbacks [3, 4]. Proactive behavior influences change in individuals, groups, and organizations and is linked to a number of positive outcomes ranging from individual performance and well-being, to group effectiveness and innovation [5–7]. Developing a good understanding of what drives individuals to be proactive is therefore important. Barack Obama’s quote implies that proactivity and affect (feeling bad or good) are intertwined. To date however, there is no compelling evidence indicating whether affect causes proactive behavior, or whether proactive behavior influences subsequent affect, and whether the role of affect in proactivity is similar for everyone or differs between individuals. This experimental study therefore investigates whether there are individual differences in the extent to which people become proactive due to affective states, and the extent to which proactive behavior regulates positive and negative affect.

Individual proactive behavior may not only enhance performance and well-being of employees in organizations, it may also facilitate positive changes in other areas, such as improving the individuals’ health. For example, [8] found that HIV patients who felt positive (optimistic) about their prospects, showed a delayed development of the disease due to proactive behaviors such as seeking information and social support, or making life-style changes. Besides pointing at health benefits of proactivity, this study indicates that positive affect makes people proactive. Similarly, organizational psychology suggests (albeit not in experimental studies) that positive affect relates to increased proactivity [9–11].

The role of negative affect is less straightforward. On the one hand, theorists assume that specific affective states that are linked to a goal can motivate proactive behavior through activating the goal to relieve the negative feeling. For example, frustration about an inefficient method may motivate someone to relieve frustration through proactivity: by initiating a better method [12]. This idea is empirically supported by longitudinal studies which show that job stressors and situational constraints at work increase proactivity [13, 14]. On the other hand, studies suggest that more general negative affect states (not linked to a specific goal) only relate to thinking about proactivity, not to implementing proactive behavior [9, 10, 15]. There thus seems to be a difference between quite specific, goal directed affective triggers and more general, core negative affect.

Although proactivity scholars state that they study the relationship between affect and proactivity, empirical work does not yet differentiate between more diffuse core affect (feeling good overall), and more specific emotions (feeling confident because of a compliment), but see [16] for conceptual work on emotions and proactivity. In the affect and emotion literature, specific emotions are seen as goal directed, whereas core-affect is less specific, does not need a target or goal, and reflects a blend of pleasantness and activation [17]. This distinction between specific emotions and unspecific affect is very important as the reason why ‘feeling good’ influences proactivity might differ from the reason why ‘feeling optimistic due to a compliment’ influences proactivity. Work related optimism likely influences proactivity through motivation, increasing energy and self-efficacy, leading people to believe that they can successfully execute the proactive behaviour. Generally feeling good likely influences proactivity cognitively, through broadening our attention focus, creating a broad thought-action repertoire that enhances proactivity [9–11, 14, 18–20].

Theoretically, positive feelings can thus enhance proactivity through a motivational pathway (through specific emotions) and a cognitive pathway (through affect). However, because empirical work on proactivity has never distinguished between positive affect and specific emotions, or between the motivational and the cognitive pathway, we currently only have a very general idea about the possible mechanisms underlying the link between positive feelings and proactivity. In order to test the impact of the cognitive pathway specifically, we focused on core affect in the current paper, to better understand *how* positive feelings enhance proactivity. We apply the mood-as-information theory [21] to test whether positive affect may enhance proactive behavior through enhancing broad and flexible cognition or whether negative affect may enhance it through enhancing more narrow and systematic cognition.

Besides the distinction between affect and emotions, there are several other reasons why it is currently not yet clear whether affect influences proactivity, and if so, how. First, the relationship between affect and proactivity is supported by mostly cross-sectional and a few longitudinal studies. Proactivity scholars thus call for experimental research [22]. Second, the reversed link (does proactivity change affect?) has hardly been examined, but see [15] for an exception). Finally, most studies use self-rated proactivity measures to investigate the relationship between affect and proactive behavior. However, those measures were originally developed to assess the trait-aspect of proactivity [23]. Particularly in the study of affect and proactivity, we think it is important to clearly distinguish between state- and trait-proactivity. Performing proactive behavior often (being a trait-proactive person), might influence the affective experience of the behavior (liking the behavior), and thus can play a role in what type of affect is a cause or a consequence of the behaviour [24]. In our study, we thus developed a new laboratory measure to assess proactive behavior, and used an existing trait measure to investigate trait-proactivity as a moderator in the affect- proactive behavior relationship.

**Fig 1:**
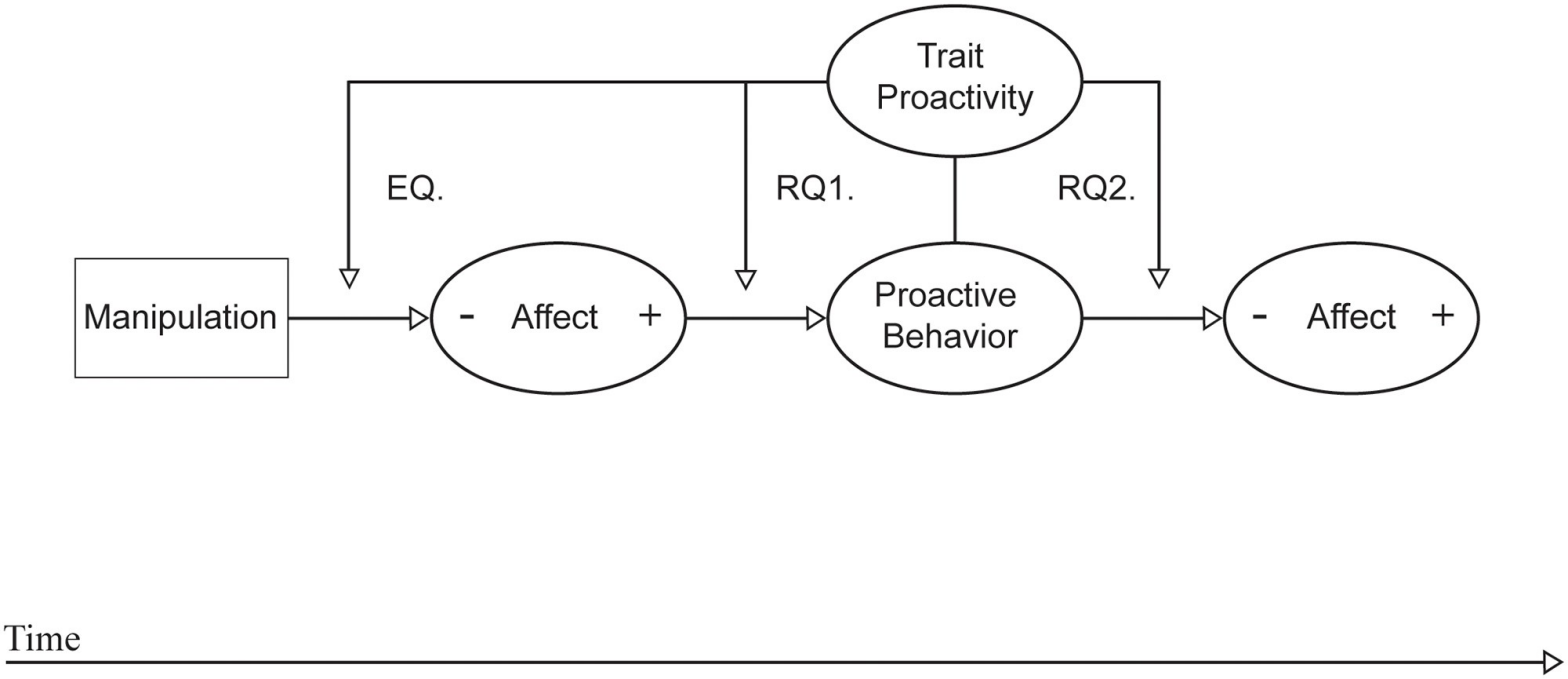
Conceptual model.

The conceptual model below (Fig 1) shows the structure of our paper. In the first part of this mixed experimental study, we investigated the causal effects of both positive *(1a)* and negative affect *(1b)* on proactive behavior between participants, and the moderating effect of trait-proactivity on the potential affective causes of proactive behavior *(1c)*. In the second part, we investigated the effect of proactive behavior on subsequent positive *(2a)* and negative *(2b)* affective changes within participants, and the moderating effect of trait-proactivity on the potential consequences of proactive behavior *(2c)*.

### Theoretical framework

#### 1. Affective influence on proactive behavior

The first aim of this experiment was to study whether and how affect influences proactive behavior. Core positive and negative activating affect stimulate behaviors through different cognitive pathways [25]. Whereas positive states facilitate global processing and broad, flexible thinking [20, 26, 27], negative affect stimulates systematic localized information processing efforts. Both these affective-cognitive pathways might influence change oriented behaviors such as proactivity. For example, creativity (the generation of novel yet appropriate ideas, e.g., [28], and problem solving, are, like proactivity, both behaviors highly focused on novelty, change, and overcoming setbacks. Previous research has shown that creativity and problem solving are facilitated by positive affect through broad, flexible thinking, *and* by negative affect through focused attention and persistence [29]. In proactivity literature however, the primary assumption is that positive affect and broad thinking enhance proactivity, not that negative affect enhances proactivity through focused attention and persistence [11].

Positive activating affect (as opposed to deactivating affect, which we do not study here) is theoretically assumed to facilitate proactivity for two reasons. First, because it enhances energy and self-efficacy (i.e., confidence that once can successfully execute the proactive behavior). Second, because it facilitates broad and flexible thinking [9–11, 14, 18]. Broad thinking should aid proactive behavior because it diversifies thought-action patterns [30], which makes change oriented behaviors more likely [9]. However, studies on proactivity and affect to date usually measure specific positive emotions at work that seem predominantly confidence-focused (i.e., feeling proud, optimistic, enthusiastic, or inspired) and that will often relate to specific events. Far fewer studies focus on core positive affect that is more clearly separated from self-efficacy. The reason why in this study, we focus solely on core positive affect, is that we wish to test only the assumption that positive affect enhances proactive behavior through broad thinking, not the assumption that positive affect enhances proactive behavior through impacting self-efficacy. In this paper, we thus focus on the affective-cognitive pathway towards proactivity.

We take the same approach with regard to negative affect. Like proactivity’s link with positive affect, the proposed and studied relationships between negative affect and proactivity to date are also primarily based on target- and task specific negative emotions. Negative affect regarding stressors at work is assumed to influence proactivity because such feelings activate people to change the status quo [10, 13, 14, 15]. When people are frustrated about constraints, for example, an inefficient filing system at their work, they can proactively prevent the future reoccurrence of this frustration by inventing a new system. Under these circumstances, proactivity thus directly targets the source of a negative emotion. What is currently still missing for negative affect as well, is research on core affect (instead of specific emotions) as this might allow for drawing further conclusions about the cognitive processes underlying affect’s influence on proactive behavior.

Although proactivity scholars typically propose that positive affect influences proactivity through broad-flexible thinking [11], we argue here that negative affect likely fosters proactivity through enhancing focused attention, systematic thinking, and persistence. On the one hand, proactive people are open to experience (advocating for the broad-flexible pathway), on the other hand, they are conscientious, which links more to a systematic-persistent approach [7, 31]. Proactive behavior is anticipatory, planned, goal directed, and persistent in the face of setbacks. Detailed, systematic-persistent thought stimulated by negative affect, might thus enhance proactivity. Previous work on creativity has shown that negative affect can enhance creativity through cognitive persistence [29]. Furthermore, although both creativity and proactive behavior are change oriented [32], proactive behavior likely requires even more persistence than creativity. Proactivity goes much beyond the generation of an idea. It also involves planning action, implementing action, and reflecting upon the action [9]. Cognitive persistence thus seems important to sustain proactive behavior beyond the mere generation of an idea to change something.

In this study, we test the causal effects of both core positive and negative activating affect on proactive behavior, in order to unravel which type of cognitive processing might be (most) important for proactivity. The literature implies that positive affect should make people more proactive because positive affect facilitates broad thinking. We propose that negative affect should make people more proactive because negative affect enhances systematic and persistent thinking. We thus test two hypotheses:

*H1a*- *Positive affect enhances proactive behavior*
*H1b*- *Negative affect enhances proactive behavior*

Affect may influence proactive behavior differently in individuals who are often proactive (trait-proactive individuals) and people who are rarely proactive (trait passive-reactive individuals). If proactivity is predominantly facilitated by one cognitive mental pathway, trait-proactive people should naturally think more in that specific way. For example, if systematic-persistent thinking enhances proactivity, trait-proactive individuals should be proactive in negative and neutral affective circumstances, because systematic-persistent thinking is likely their cognitive default. Proactive people may not need negative affect because they are able to focus their attention systematically and persist under neutral circumstances, whereas passive-reactive people should be proactive only when experiencing negative affect because they need the persistent-systematic activation. If broad-flexible thinking is most important, proactive individuals should be most proactive in positive and neutral affective circumstances (as they are naturally in the preferred broad-flexible state), whereas passive-reactive individuals should be proactive only when experiencing positive affect (needing broad-flexible activation). If either of these two cognitive-affective pathways enhances proactive behavior more than the other, that effect should be amplified in passive-reactive individuals. The affect-type (positive or negative) that increases proactivity of passive-reactive individuals should thus indicate which cognitive pathway most likely facilitates proactive behavior. Because we have proposed that both positive affect *(H1a)* and negative affect *(H1b)* may enhance proactive behavior, we have not predefined the direction of our moderation hypothesis. We thus test the following hypothesis:

*H1c*- *Trait-proactivity moderates the influence of affect on proactive behavior*.

#### 2. Affective consequences of proactive behavior

The second aim of this experiment was to investigate affect regulation effects of proactivity, by assessing how people feel after showing proactive behavior. In a diary study, [15]) found that people report to feel positive after engaging in proactive behavior. Perhaps the positive relationships between positive affective states and proactive behavior that are usually found in the literature can be (at least partly) explained by the idea that proactive behavior makes people feel good, instead of the other way around. Although current theory points at positive affect as the cause of proactivity, we argue that positive affect also forms a possible consequence of proactivity. Proactive behavior is about influencing and changing one’s environment: a type of control likely to enhance positive feelings and energy levels and decrease negative feelings when exercised. In this sense, proactive behavior could play an important role in affect regulation (the extent to which people are able to ‘manage’ their feelings, see [33]. We thus test the following hypotheses:

*H2a*- *Proactive behavior enhances positive affect*.
*H2b*- *Proactive behavior diminishes negative affect*.

The Emotions as Feedback Theory [24] proposes that behavior is executed to pursue future (positive) affective states that were previously linked to such behavior. If proactive behavior enhances positive affect, as proposed in *H2a*, those individuals who have experienced this may (either explicitly or implicitly) use proactive behavior to enhance positive affect. Since trait-proactive individuals are likely more familiar with the affective consequences of proactivity, for them, anticipated positive affect could be a motivator for proactive behavior. This may result in decreased proactive behavior in this group when they are already feeling good (for example, when positive affect is induced) as there is no need for increasing positive feelings. Another consequence of trait-proactivity is that because people perform proactive behavior often, they presumably enjoy it more than passive-reactive individuals and hence, experience a stronger increase in positive affect after they engage in proactive behavior. We thus test the following hypothesis:

*H2c* - *Trait-proactivity moderates the relationship between proactive behavior and subsequent changes in affect*, *such that trait-proactive individuals report stronger increases in positive affect after executing proactive behavior than trait passive-reactive individuals*.

#### 3. The influence of trait proactivity on affective sensitivity

Finally, we explored another potential affective difference between proactive and passive- reactive individuals. If, as proposed above, particularly trait-proactive individuals use proactive behavior to enhance positive affect, there should be an affective reason why they need to enhance positive feelings. One reason could be that proactive individuals are affectively more sensitive than passive-reactive individuals and that proactive behavior, to them, is a way to cope with their affective sensitivity. Trait-proactivity is related to creativity [34], which relates to negative affective vulnerability, self-reflective thought, and cognitive elaboration [35, 36]. We test whether something similar is the case for trait-proactive individuals. If they are sensitive to negative affective stimuli, they may have learned to deal with their sensitivity by up-regulating positive affect through engaging in proactive behaviors, thus ‘managing’ their affective states by taking control in another area (in which one is proactive). We propose that proactive individuals are generally more sensitive to negative affective stimulation than passive-reactive individuals, and that proactive behavior, to them, is a way to cope with their affective sensitivity. We explore this possibility of a differential trait-proactivity related sensitivity to affective stimulation by measuring both reported affect and physiological activation in response to our affective manipulations and relating these affective responses to trait-proactivity. We thus test the following hypothesis:

*H3—*Proactive individuals show stronger affective and physiological reactions to negative affective stimuli than passive-reactive individuals.

### Present research design

**Fig 2:**
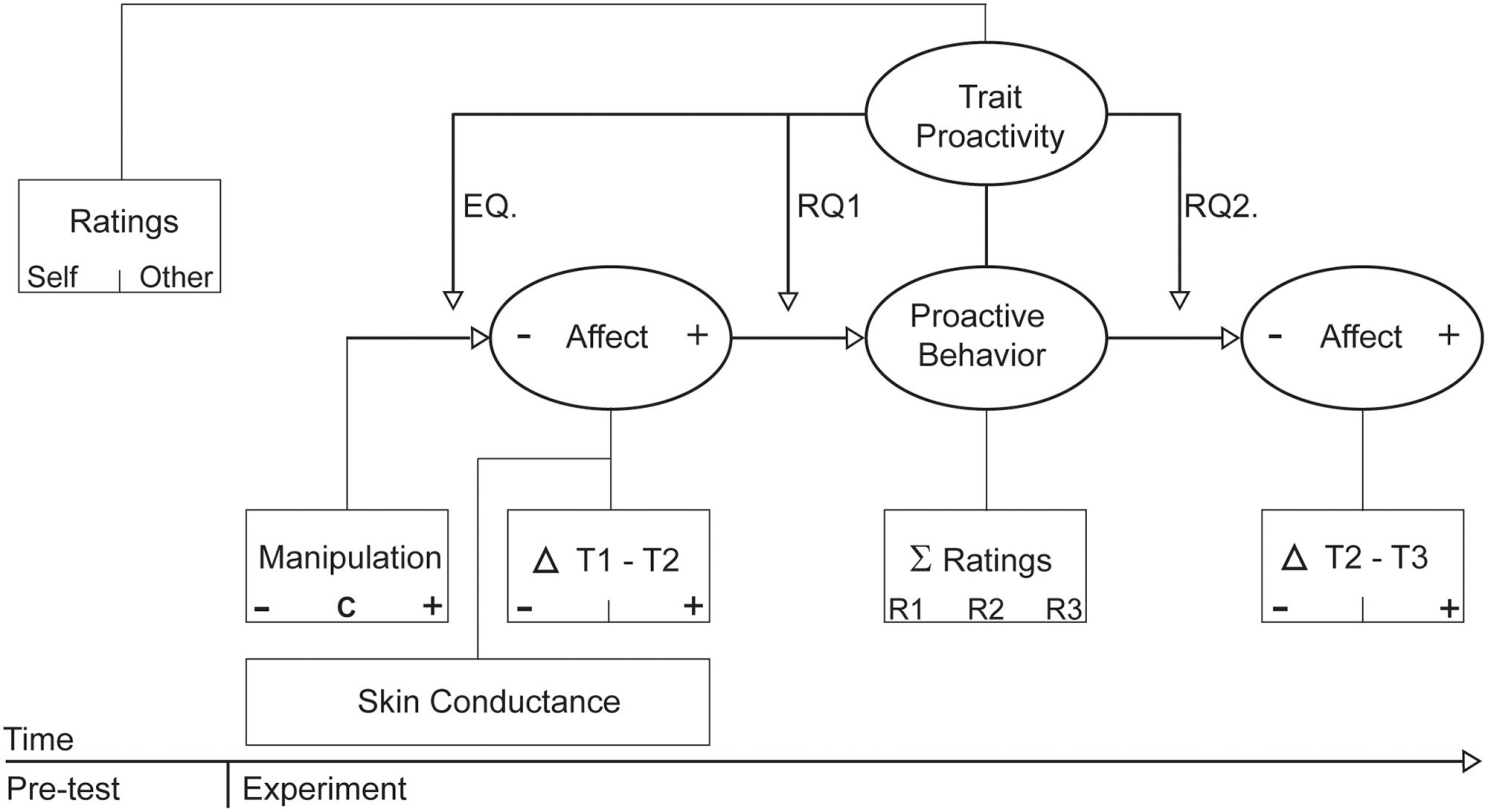
Study design.

We conducted an experimental study on proactive behavior testing both the effect of positive and negative affect on proactivity (see Fig 2 for a visual overview). We randomly assigned participants to one of three conditions (negative, neutral, or positive), in which we manipulated core affect through a combination of pictures and music. We repeatedly measured self-reported affect and physiological measures of arousal during manipulation to assess sensitivity to those manipulations. We designed a lab measure of proactive behavior to assess which type of affect manipulation influenced proactivity. We further tested whether proactive behavior in the lab predicted changes in positive and negative affect over time, using repeated measures of positive and negative affect before and after participants showed (or did not show, as proactivity was voluntary) proactive behavior. Trait-proactivity was measured three weeks prior to the experiment to make sure the measurement of the moderator was clearly separated from all other measures in the experiment. In summary, we expected trait-proactivity to moderate *1)* which type of affect influences proactivity, *2)* what type of affect results from proactivity, and *3)* how people affectively respond to the negative affect manipulation.

## Methods

### Procedure, ethics, and sample

#### Procedure

Three weeks before the experiment started, students completed an online survey (in Qualtrics) in the classroom measuring trait-proactivity and they sent a duplicate trait-questionnaire link to their best friend or significant other. They could win a raffle reward of €100,—for participating in this pre-test. Later, upon arrival in the lab, students were welcomed by the experimenters in pairs because they needed to believe they were interacting with each other. Both participants gave written informed consent and were then installed in separate cubicles, where they played two memory games on an iPad to mask the purpose of the study (the title of the study was ‘Memory Games’). Next, participants were attached to the skin conductance device and positioned in front of the computer screen with headphones. We measured baseline affect (T1), followed by the 5-minute affect manipulation, the affect manipulation check (T2), and on-screen instructions about the leader-follower structure of the proactive task. Next, they were disconnected from the skin conductance device and experimenters brought printed information for the proactivity task. On average, the task lasted 8.18 minutes (*SD* = 2.23). The proactivity task was followed by the final affect measurement (T3) and an exit interview, where we checked whether participants were naïve to the study purpose and design. Afterwards, participants were debriefed, thanked and paid (€10, -).

#### Ethics

This study was approved by the Ethics Review Board of the University of Amsterdam, Faculty of Social and Behavioural Sciences on March 31st, 2015, and conducted in April and May of 2015, according to the principles expressed in the Declaration of Helsinki. Before the study, participants were explicitly informed (both through reading the information brochure and by the experimenter) that this experiment involved looking at emotional content that could be experienced as too intense, and that were free to terminate the experiment at any moment should they feel in any way too uncomfortable (4 participants in the negative affect condition chose this option). Informed consent (written) was obtained from all participants. All data was examined anonymously. Participants were aware that they could terminate their participation at any time, and retract their responses within 7 days after participation. Participants were first paid, and then debriefed. The data were collected over a period of 4 weeks. Below, we provide an overview of our methods. A more extensive description of the composition of the proactive task can be found here: dx.doi.org/10.17504/protocols.io.tubensn.

#### Sample

Students recruited in the classroom were pre-tested on trait-proactivity (*N* = 580) and asked to voluntarily participate in the experimental study called ‘Memory Games’ (32% participated). Students (*N* = 180, *M*_age_ = 22.39, *SD*_age_ = 5.30, 67% female, largest groups: 29% business and economics, 25% psychology) in different years of enrolment (*M* = 2.19, *SD* = 1.30) participated in the experiment for a monetary reward (€10, -). We targeted 180 participants (see stopping-rule in results section). Participants were randomly distributed across affect conditions (*N*negative = 58, *N*control = 60, *N*positive = 62).

### Manipulations, measures, and tasks

#### Affect manipulation

Activating positive, negative, and neutral (control) affect was induced between groups. We used simultaneous visual and auditory stimulation since Baumgartner, Esslen, and Jäncke [37] showed that emotional experience evoked by affective pictures is enhanced through music. Three different musical excerpts were played through headphones: John Adam’s ‘Common tones in simple time’ in the neutral affect (control) condition, Clint Mansell’s movie soundtrack ‘Requiem for a dream’ in the negative affect condition, and a compilation of Shostakovich’s 1st and 2nd ballet and jazz suites in the positive affect condition (see S1 Appendix for details). Visual material was selected from the well-validated NAPS [38] and IAPS [39, 40] picture databases. We selected 20 pictures per condition based on equal valence and arousal for men and women. Pictures in the positive (i.e., 3 puppies, a snowboarder with lush mountains in the background), neutral (a woman with neutral facial expression, a bag of potatoes), and negative condition (a car- crash, a child with an amputated leg) differed in terms of valence (all *p* < .001). Social- versus object-type pictures were evenly distributed across conditions. Pictures were presented for 5000 milliseconds (ms), followed by jittered inter-stimulus intervals, ranging between 5500 and 7500 ms before the next stimulus presentation.

#### Independent variable and moderator: Trait-proactivity

To ensure that trait-proactivity–behavior relationships in the experiment would not be confounded by temporal ‘activation’ of proactivity, we measured the trait three weeks prior to the experiment. Trait-proactivity was measured with self-ratings (*α* = .752) and ratings by romantic partners or best friends (*α* = .813) of the European personal initiative scale that is widely used in proactivity research as a valid scale to measure trait-proactivity [24, 41]. More information about this scale compared to the American proactive personality scale and reasoning behind our choice can be found in the S1 Appendix. Both scales consisted of 8 items (1–7 Likert), examples are: ‘I/My partner take(s) initiative immediately even when others do not’ and ‘I/My partner actively attack(s) problems’. Self- and other ratings were correlated, *r* (161) = .432, *p* < .001. In comparison, Fay and Frese [24] report a correlation of *r* (220) = .350, *p* < .01. In line with earlier work from these authors, self- and other ratings were averaged into one variable, trait-proactivity (*α* = .838).

#### Reported positive and negative affect

We measured activating positive and negative affect 3 times: before the affect manipulation (T1), after the manipulation (T2), and after the proactivity task (T3). Affect changes during the manipulation were reflected in individual changes from T1 to T2, affect changes during the proactive task were reflected in individual changes from T2 to T3. We used 20 items adopted from Hess and Blairy [42] that were designed to capture the valence (‘I feel bad’) and activation (‘I feel tense’) aspects of negative affect, as well as the valence (‘I feel pleasant’) and activation (‘I feel energized’) aspects of positive affect. Affect items were displayed in the middle of the computer screen. Participants indicated to what extent the items reflected their current state on a continuous slider ranging from 0 (not at all) to 100 (strongly). The positive affective scale was highly reliable across all 3 time points (9 items, *α*^*T1*^ = .904, *α*^*T2*^ = .943, *α*^*T3*^ = .912) as was the negative activating affective scale (11 items, *α*^*T1*^ = .851, *α*^*T2*^ = .927, *α*^*T3*^ = .889). All items and factor loadings can be found in the supporting information (S1 Appendix, Tables A and B).

#### Skin conductance response to manipulation

As an index of physiological reactivity in response to the affect manipulations we measured event related electrodermal activity [40]. This measure has been used previously to study the relationship between personality characteristics and physiological reactivity to affective stimuli [43]. We attached two 20 mm by 16 mm Ag/AgCl electrodes to the medial phalanges of the third and fourth fingers of the participant’s left hand. The electrodes were connected to an input device with a sine shaped excitation voltage of 1 Vpp at 50 Hz (derived from the mains frequency). A signal-conditioning amplifier converted the signal into a linear output range of 0 uS to 100 uS (measured as a range of –10 to þ10 V). The analogue output was then digitized at 250 samples per second by a 16-bit AD-converter (Keithley Instruments KPCI-3107). Skin conductance responses (SCR) were calculated during the full duration of each picture (5000 ms) by searching for troughs and peaks in the signal, with a minimum magnitude of 0.05 uS and a minimum trough-to-peak length of 100 ms. In order to reduce the impact of extreme values and for normalization purposes, all data was square root transformed [44, 45]. Analyses were performed on the square root SCR magnitude (i.e., calculated across all trials, including zero or no response trials, Payne et al., 2016) averaged across the different pictures presented during the manipulation.

#### Proactive behavior

Following the manipulation check, we assessed participants’ proactive behavior during a team task with an updated version of the proactivity task designed by Wolsink et al. [46]. All teams were composed of one follower (the participant) and one leader. We emphasized that the leader was expected to be proactive, take the lead, start the conversation, solve the problem and justify his/her choices after the task, whereas the follower had the opportunity to be proactive, but was not explicitly expected to do so. We told all participants that they were randomly assigned to the follower role and that the other participant was assigned to the leader role. Participants thus thought they were communicating with the other participant (the one elected team leader), while in fact the leader was simulated by the computer.

The leader’s goal was to choose the best new dean for the faculty from three different candidates. In order to select the best dean, both the participant and the leader received hard-copy information about three dean-candidates (A, B, and C). The information about these candidates, however, was not identical for the leader and the participant and should result in a leader preference for candidate B and a participant preference for candidate A or C (a pilot study showed 100% initial participant preference for A or C, i.e., no participant preferred B). This information conflict served as an opportunity for the participant to become proactive and take initiative to try to change the leader’s wrong decision for the benefit of the team, even though he/she was not required to do so.

We designed the task to reflect persistent initiative and challenging behavior, i.e. proactive behavior. The initiative component was measured first. When the participants received the candidate profiles, they had 6 minutes to read it and take the initiative to start a conversation with the leader, even though they were not responsible for the decision. All initiatives from participants were textually recorded, and later coded by three independent raters (*ICC* = .749): 0 = no initiative, 1 = initiative but without extensive content (example: ‘what candidate do you prefer?’), 2 = initiative with extensive content (example: ‘I advise to go for candidate A since this person is most passionate about the connection with the labor market and is trying to make sure that knowledge and demand adjoin one another.’). Ratings (range 0–2) of all judges were summed up (range 0–6).

Next, we measured challenging behavior. After 6 minutes, regardless of whether the participant had taken initiative or not, the (computer-simulated) leader ‘took the lead’, and said: ‘Based on my information here, it is obvious that we should go for option B’. To solve the information conflict (participants’ information suggested A or C was best), the participant could now either comply with the leader or challenge the decision of the leader. The degree of challenge was again rated by 3 judges (*ICC* = .912): 0 = no challenge, 1 = challenge without extensive content (example: ‘Our information differs a little, but A is still the best’), 2 = challenge with extensive content (example: ‘C and A are comparable, but C seems more ready to cooperate and listen to other people than A. B had the most negative attributes as well as the most extreme. C is my choice, because he has least negative attributes and is willing to listen to student councils’). Ratings (range 0–2) of all judges were again added up (range 0–6).

To reflect persistent, goal directed proactive behavior across time, we created our dependent variable ‘proactive behavior’ as the sum score of the initiative ratings and challenge ratings of all three judges (range 0–12). Those who were thus most persistent in their proactivity throughout the experiment showed both extensive initiative and extensively challenged the leader, thereby changing the outcomes for the team.

## Results

### Participants, stopping rule, and exclusion criteria

Since to our knowledge this is the first experiment on affect and proactive behavior, there was no prior data for a power analysis, and so we set our stopping rule at 180 participants to approach a number of 30 participants per cell if we were to eventually split the trait proactivity in a high and low group (which we only did for graphical purposes), resulting in 3 affect groups * 2 trait groups [47]. Eighteen participants were excluded from analyses. We lost most participants (*N* = 10) due to technical problems with the skin conductance device or because participants in the negative condition terminated the experiment because they were too affected by the stimuli. We purposely removed the other eight (*N* = 8) participants. Some (*N* = 3) because they were aware of the purpose of the experiment: in the open question at the end, these three participants mentioned proactive behavior/taking initiative as the dependent variable of the study and indicated a relationship with mood/emotions/affect. Others were extreme outliers (*N* = 2) on age (> 8 *SD* above the mean) and physiological response to the neutral stimuli (> 5 *SD* above the mean). Three people (*N* = 3) were excluded because we could not reliably measure their proactivity: one was not native and responded in English to Dutch language stimuli, one could not type during the initiative phase, and one did not understand the instructions. Complete data of the remaining 162 participants were spread relatively equally over the three affect conditions (see Descriptives Table 1). There were no differences between affect conditions on trait-proactivity, baseline negative affect, baseline positive affect, and age (all *F’*s < 1, all *p*’s >.250) and gender was equally represented in all conditions *χ*^*2*^ (2, *N* = 162) = 1.93, *p >* .250).

**Table 1:** Participants and descriptives.

Affect Condition		Proactive Behavior		Trait Proactivity		Baseline Negative Affect		Baseline Positive Affect		Age		Gender	
	*N*	*M*	*se*	*M*	*se*	*M*	*se*	*M*	*se*	*M*	*se*	*F*	*M*
Negative	**54**	6,91	0,43	5,08	,08	21,15	1,82	68,59	1,76	22,36	0,87	36	18
Control	**50**	5,96	0,45	4,93	,10	19,45	1,80	66,42	2,32	22,39	0,48	34	16
Positive	**58**	5,72	0,41	5,05	,09	21,07	1,50	66,56	1,89	22,21	0,33	45	13
Total	**162**	6,19	0,43	5,02	,05	20,59	,98	67,19	1,14	22,31	0,34	115	47

### Manipulation checks

We used a 3 (condition) * 2 level (baseline affect—affect after the manipulation) repeated measures ANOVA to test whether participants showed positive and negative affect changes due to the manipulation. As expected, affect condition influenced within person changes (ΔT1 –T2) in negative affect, *F* (2, 159) = 80.19, *p* < .001, *η*^*2*^ = .502, and positive affect, *F* (2,159) = 40.99, *p* < .001, *η*^*2*^ = .340. Participants felt worse in the negative condition. Compared to the neutral condition (*M*_*Δ negative affect*_ = -1.05, *se* = .97, *CI*_*95*_ = {-3.19, 0.85 }, *M*_*Δ positive affect*_ = -2.07, *se* = 1.12, *CI*_*95*_ = {-4.39, 0.13 },), participants in the negative condition reported increased negative affect (*M*_*Δ negative affect*_ = 17.08, *se* = 1.89, *CI*_*95*_ = {13.43, 20.93}), *F* (1, 102) = 69.99, *p* < .001, *η*^*2*^ = .407, and decreased positive affect (*M*_*Δ positive affect*_ = -14.72, *se* = 1.95, *CI*_*95*_ = {-18.49, -11.06}), *F* (1,102) = 30.99, *p* < .001, *η*^*2*^ = .233. In the positive condition, participants reported decreased negative affect (*M*_*Δ negative affect*_ = -4.25, *se* = .76, *CI*_*95*_ = {-5.72, -2.72}), *F* (1,106) = 6.85, *p* = .01, *η*^*2*^ = .061, and increased positive affect (*M*_*Δ positive affect*_ = 3.41, *se* = 1.24, *CI*_*95*_ = {0.98, 5.77}), *F* (1,106) = 10.96, *p* = .001, *η*^*2*^ = .094, as compared to the neutral condition. We thus conclude that our affect manipulations worked: the positive affect condition enhanced positive affect, and the negative affect condition enhanced negative affect.

The physiological data showed that the reaction to the negative condition was stronger than the reaction to the positive and neutral condition. Participants demonstrated a higher response magnitude (SCR) to the images in the negative condition (*M*_*magnitude*_ = .22, *se* = .02, *CI*_*95*_ = {0.15, 0.24 }) than in the neutral condition (*M*_*magnitude*_ = .12, *se* = .02, *CI*_*95*_ = {0.09, 0.16}), *F* (1,102) = 5.86, *p* = .017, *η*^*2*^ = .054, but between the positive (*M*_*magnitude*_ = .13, *se* = .02, *CI*_*95*_ = {0.10, 0.16}) and neutral condition, there was no magnitude difference, *F* (1,106) = 0.03, *p* > .250, *η*^*2*^ *<* .001.

### Main results

#### Analyses

All three proposed interaction effects were tested with ANCOVA’s, where trait-proactivity was modelled as a continuous variable to ensure robustness of the effects. Any covariate used in these analyses was Z-standardized because unstandardized covariates can severely change main effects, especially in repeated measures ANOVA’s [48]. We report significant results at *α* = .05 level. Only for explanatory purposes and graphical representation, we created two groups of participants using a mean-split for trait-proactivity (interaction-effects of continuous *and* group- variables are summarized in S1 Appendix, Table C). We report separate results for proactive individuals (those who score above the trait-proactivity mean) and passive-reactive individuals (those who score below the trait-proactivity mean) to specify the directions of the interaction affects.

#### 1: When affect influences proactive behavior

Our first aim was to test whether our affect manipulation influenced proactive behavior, and whether these effects differed for proactive individuals versus passive-reactive individuals. We tested this with a 3 (affect condition) * continuous (trait-proactivity) between subjects ANCOVA with proactive behavior as the dependent variable. Regarding hypotheses *1a* (positive affect influences proactive behavior) and *1b* (negative affect influences proactive behavior), we did not find a main effect of affect condition on proactive behavior, *F* (2, 161) = 2.04, *p* = .13, *η*^*2*^ = .025. Proactive behavior in the negative (*M*_*proactive behavior*_ = 6.91, se = .42, *CI*_*95*_ = {6.06, 7.74}) and positive (*M*_*proactive behavior*_ = 5.76, se = .41, *CI*_*95*_ = {4.96, 6.57}) affect condition did not significantly differ from the control condition (*M*_*proactive behavior*_ = 5.86, se = .44, *CI*_*95*_ = {4.99, 6.73}), Bonferroni corrected. However, proactive behavior was highest in the negative affect condition (*M* = 6.91), and when comparing the negative to the combined neutral and positive conditions (*M*_*proactive behavior*_ = 5.81, se = .30, *CI*_*95*_ = {5.22, 6.40}), the difference was significant, *F* (1, 161) = 4.20, *p* = .042, *η*^*2*^ = .026. These results thus show more support for the hypothesis that negative affect enhances proactive behavior (*H1b*), than for the hypothesis that positive affect enhances proactive behavior (*H1a*).

**Fig 3:**
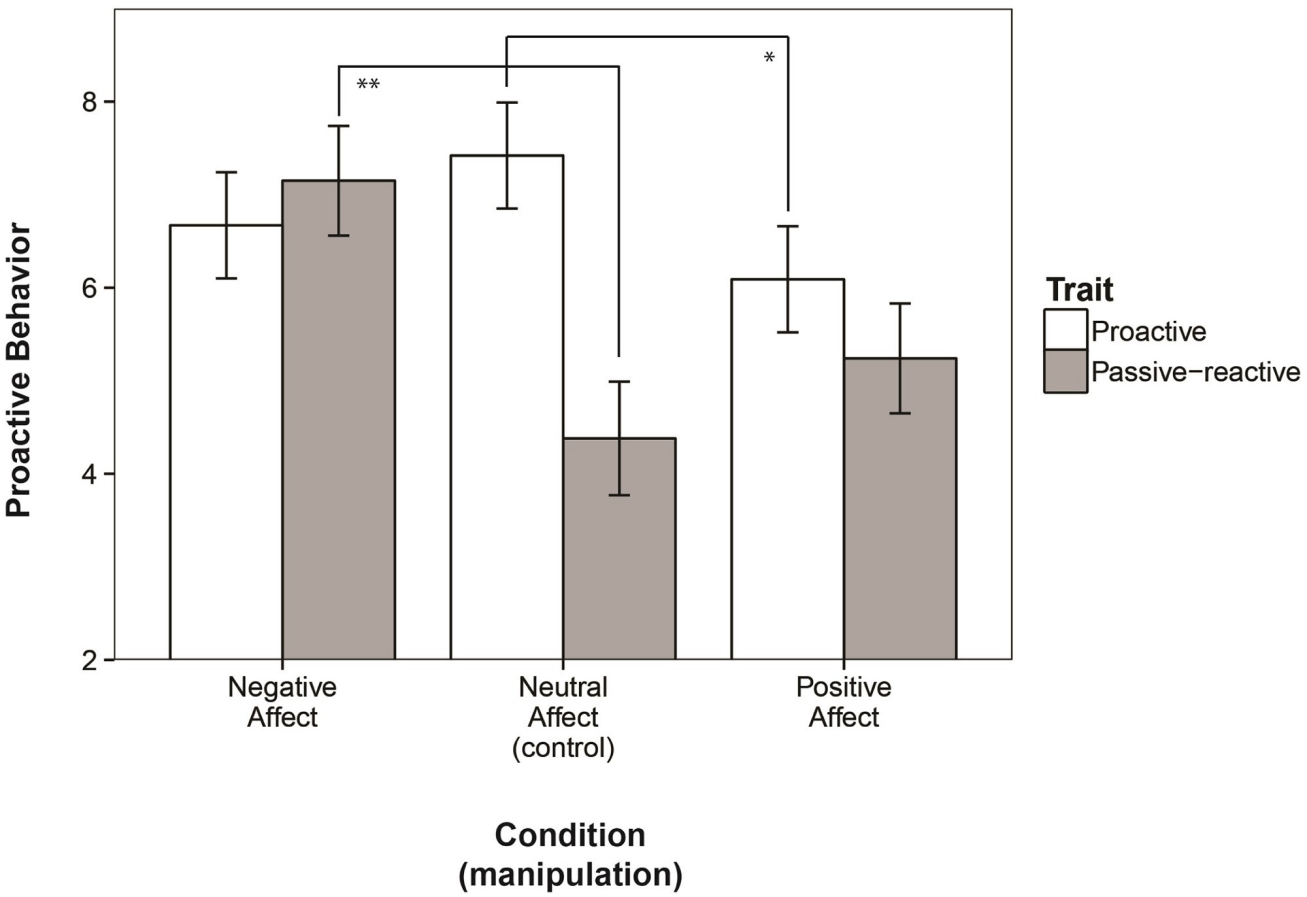
Effects of affect conditions on proactive behavior, moderated by trait-proactivity.

Next, we hypothesized that trait-proactivity would moderate the influence of affect on proactive behavior (*H1c*). We found the expected interaction between trait-proactivity and affect condition, *F* (2, 161) = 3.13, *p* = .047, *η*^*2*^ = .039. Fig 3 shows that passive-reactive individuals were significantly more proactive in the negative affect condition (*M*_*proactive behavior*_ = 7.15, *se* = .59, *CI*_*95*_ = {5.99, 8.31}) than in the neutral condition (*M*_*proactive behavior*_ = 4.38, *se* = .61, *CI*_*95*_ = {3.11, 5.64}), *F* (1, 76) = 11.871, *p* = .001, *η*^*2*^ = .135. In contrast, proactive individuals were not. They were equally proactive in the negative affect condition (*M*_*proactive behavior*_ = 6.67, *se* = .59, *CI*_*95*_ = {5.51, 7.83}) and the neutral condition (*M*_*proactive behavior*_ = 7.42, *se* = .57, *CI*_*95*_ = {6.24, 8.61}), *F* (1, 79) = .527, *p* = .470, *η*^*2*^ = .007. This indicates that for passive-reactive individuals, a persistence-stimulating negative affective state stimulates proactivity, whereas for proactive people, a negative affective state is not necessary for proactivity. All main and interaction effects are summarized in Table 2.

**Table 2:** Main and interaction effects of affect condition * trait-proactivity on proactive behavior.

		Affect Condition					
Dependent	Moderator	Negative		Neutral (control)		Positive	
Proactive Behavior	Trait-proactivity	*M*	*se*	*M*	*se*	*M*	*se*
Interaction (*H1c*)	Proactive	6.67	0.57	7.42	0.57	6.09*	0.57
	Passive-reactive	7.15**	0.59	4.38	0.61	5.24	0.59
Main effects (*H1ab*)	Total	6.91	0.41	5.96	0.47	5.72	0.42

Note.

* *p* < .05,

** *p* < .01.

Significant compared to control condition

Interestingly, passive-reactive individuals did not only show more proactive behavior in a negative state, proactive individuals also showed less proactive behavior in a positive state. When comparing the positive affective states to neutral states for both trait groups, in the positive affect condition (*M*_*proactive behavior*_ = 6.09, *se* = .53, *CI*_*95*_ = {5.04, 7.14}), proactive participants behaved less proactively than in the neutral condition (*M* = 7.42), *F* (1, 83) = 5.42, *p* = .022, *η*^*2*^ = .062. This implies that broad and flexible thinking, which is stimulated by positive affect, does not necessarily enhance proactive behavior, especially not in trait-proactive people. Passive-reactive participants were equally (un)proactive in neutral (*M* = 4.38) and positive affective states (*M*_*proactive behavior*_ = 5.24, *se* = .61, *CI*_*95*_ = {4.03, 6.45}), *F* (1, 81) = 3.19, *p* = .078, *η*^*2*^ = .039.

In summary, the results show that proactive individuals show less proactive behavior when they are in a positive affective state than in a neutral state, whereas passive-reactive individuals show more proactive behavior when they are in a negative affective state than in a neutral state. These results do not support *H1a*, which stated that positive affect enhances proactive behavior. Rather, they support *H1c*, stating that affects’ influence on proactive behavior depends on trait-proactivity, and *H1b*, that negative affect enhances proactive behavior, albeit only in passive-reactive individuals. This supports the idea that negative affect and persistent-systematic thought are more important for proactive behavior than positive affect and broad-flexible thought.

#### 2: When proactive behavior influences affect

Our second aim was to investigate the effect of proactive behavior on subsequent affect (*H2ab*), and assess the difference between trait-proactive individuals and passive-reactive individuals in this respect (*H2c*). We used a repeated measures ANCOVA with continuous (covariates) independents proactive behavior * trait-proactivity, and 2 levels of positive and negative affect (affect before- and after the proactivity task) as dependent variables. All covariates were Z-standardized because unstandardized between-participant covariates can substantially affect within- participant main effects [48]. We controlled for affect condition because the condition showed to influence both proactive behavior and affect, and for time on the proactive task because the more proactive participants were, likely, the more time they spent on the proactive task. This means that the effect of proactivity on the regulation of affect could just be a mere effect of time spent on the task, rather than the proactive behavior driving the effect. We therefore controlled for time, even though our results do not change if time on task is not included in the analysis. First, we hypothesized that proactive behavior enhances positive affect (*H2a*). As expected, there was a main effect of proactive behavior on positive affect, *F* (1,154) = 7.91, *p* = .008, *η*^*2*^ = .045, indicating that proactive behavior makes people feel better. We did not however, find a significant main effect of proactive behavior on negative affect *F* (1,154) = .460, *p* = .499, *η*^*2*^ = .003. This supports the hypothesis that proactive behavior enhances positive affect (*H2a*), but not the hypothesis that proactive behavior diminishes negative affect (*H2b*).

**Fig 4:**
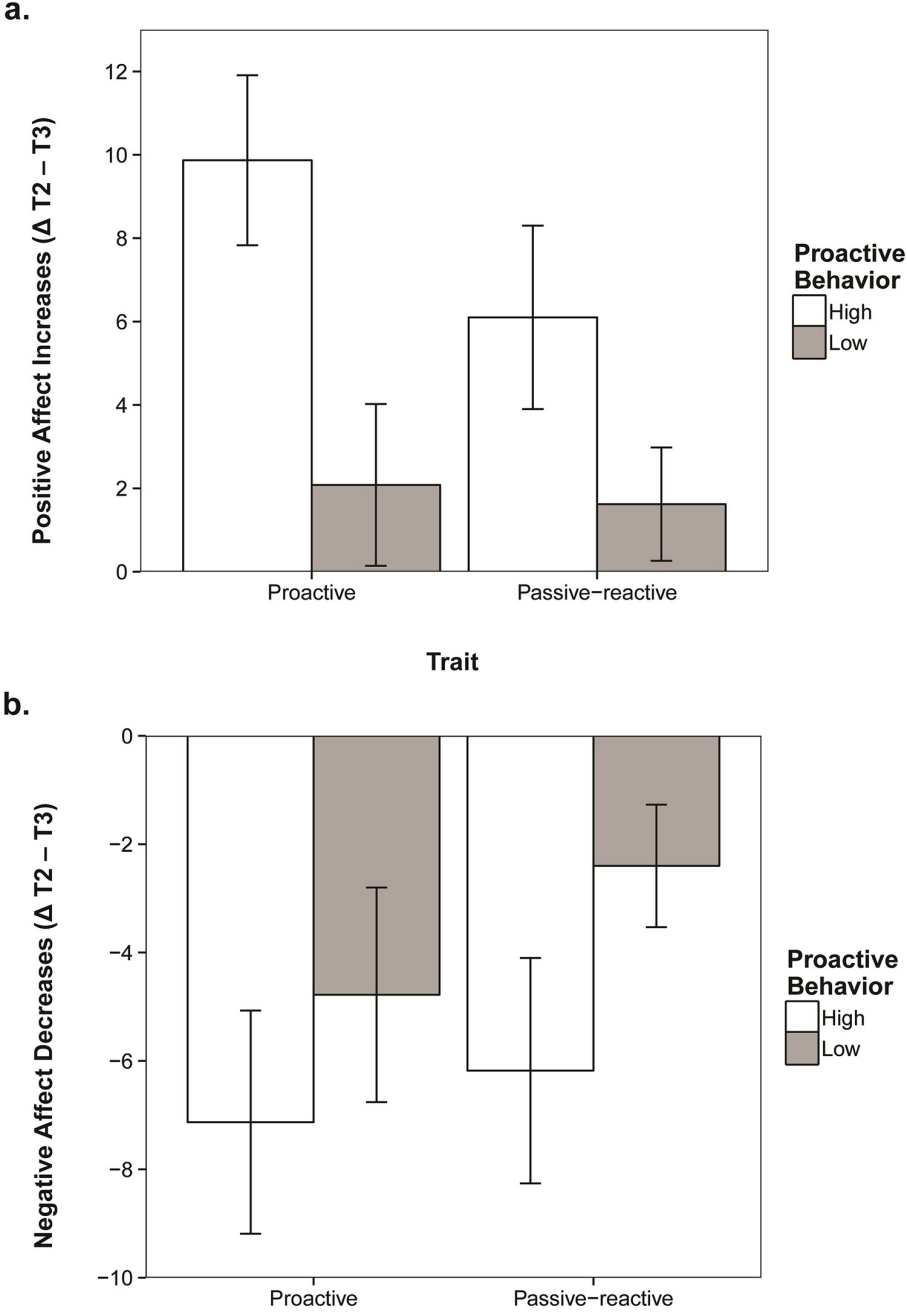
Interaction of proactive behavior and trait-proactivity. Groups (Trait and Behavior high / low) are based on mean-splits. Dependent variables are **(a)** positive affect increases after the proactivity task (difference between T2 and T3) and **(b)** negative affect decreases after the proactivity task (difference between T2 and T3).

Next, we hypothesized that trait-proactivity would moderate the regulatory effects of proactive behavior on affect *(H2c)*. In line with hypothesis *H2c*, we found a trait * proactive behavior interaction for individual positive affect increases (Δ T2 –T3), *F* (1, 154) = 5.49, *p* = .021 *η*^*2*^ = .034. Fig 4 and Table 3 show that proactive individuals who showed highly proactive behavior reported stronger positive affect increases (*M*_*Δ positive affect*_ = 9.75, *se* = 2.05, *CI*_*95*_ = {5.68, 13.83},) than proactive individuals who showed little proactivity (*M*_*Δ positive affect*_ = 2.19, *se* = 2.00, *CI*_*95*_ = {-1.79, 6.17}), *F* (1, 83) = 6.82, *p* = .011 *η*^*2*^ = .076. In the passive-reactive group, the pattern was similar but the difference in positive affect increases between those who were highly and those who were little proactive was not significant, *F* (1,72) = 1.48, *p* = .23, *η*^*2*^ = .020. Summarizing the results for positive affect after proactive behavior: participants generally felt better after proactive behavior, but positive affect increases due to proactivity were stronger for trait-proactive participants. This pattern of results was expected and hypothesized. However, we also found something unexpected when looking at changes in negative affect.

**Table 3:** Main effects and trait-proactivity * proactive behavior interactions on changes in affect from before (T2) to after (T3) the proactive task.

		Proactive behavior			
Dependent	Moderator	Low		High	
Affective Changes	Trait Proactivity	*M*	*se*	*M*	*se*
Negative Affect	Proactive	-4.65	2.03	-7.26	2.08
	Passive-reactive	-2.11	1.21	-6.22*	1.91
	Total (main effect)	-3.47	1.09	-6.80	1.52
Positive Affect	Proactive	2.19	2.00	9.75**	2.05
	Passive-reactive	1.87	1.37	5.00	2.16
	Total (main effect)	1.82	1.14	8.57***	1.55

Note.

* *p* < .05,

** *p* < .01, low proactive behavior compared to high proactive behavior.

'- ' reflects affective decreases, otherwise: affective increases. Reported values are the average individual differences between T2 and T3 affect.

Similar to the previous analysis, we also found an interaction effect between trait-proactivity and proactive behavior (*H2c*) for negative affect decreases (Δ T2 –T3), *F* (1, 154) = 5.01, *p* = .027 *η*^*2*^ = .032, but the pattern was reversed. Only the passive-reactive group showed a negative affect decrease due to proactive behavior. Passive-reactive individuals who were proactive (*M*_*Δ negative affect*_ = -7.26, *se* = 2.08, *CI*_*95*_ = {-10.03, -2.42}) showed stronger decreases in negative affect than passive-reactive individuals who were not proactive (*M*_*Δ negative affect*_ = -2.11, *se* = 1.21, *CI*_*95*_ = {-4.52, 0.30}), *F*_*median* -split_ (1, 80) = 5.609, *p* = .020, *η*^*2*^ = .066, *F*_*mean* -split_ (1, 74) = 3.20, *p* = .078, *η*^*2*^ = .043. Although the effect is not robust across group-splits (mean vs. median), indicating it is small, the significant interaction between the continuous measures of proactive behavior and trait-proactivity does suggest that proactive behavior down-regulated negative affect most for passive-reactive individuals.

#### 3. The influence of trait proactivity on affective sensitivity

Since we expected trait-proactivity to moderate the relationship between affect and proactive behavior, we implicitly assumed that proactive individuals might react differently to affective triggers than their passive-reactive counterparts *(H3)*. Moreover, as proactive individuals tend to be more ‘active’ than passive reactive individuals, we wanted to explore whether they are more easily ‘activated’ than passive-reactive individuals in terms of physiology. Specifically, based on the aforementioned work on creativity [35, 36], we suggested that trait-proactive individuals may react more strongly to negative affective triggers. We thus explored whether proactive and passive-reactive individuals showed different reactions to our affect-manipulations. To test this, we used a repeated measures ANCOVA with affect-condition (factor) * trait-proactivity (covariate) as independents, and 2 levels of positive and negative affect (baseline affect–affect after manipulation) as dependent variables. All means and standard errors are reported in Table 3 and Fig 4.

In support of *H3*, we found an interaction between affect condition and trait-proactivity on reported negative affect changes, *F* (2, 161) = 4.31, *p* = .015, *η*^*2*^ = .052, and positive affect changes after the manipulation, *F* (2, 161) = 5.20, *p* = .006, *η*^*2*^ = .063. Although both trait-groups reported increased negative affect and decreased positive affect in the negative condition (compared to neutral, all *F*’s > 15.85, all *p*’s < .001), proactive individuals showed the strongest affective reactions to the negative condition. Proactive individuals reported stronger negative affect increases (*M*_*Δnegative affect*_ = 22.60, *se* = 2.88, *CI*_*95*_ = {16.69, 28.51}) than passive-reactives (*M*_*Δnegative affect*_ = 11.56, *se* = 1.94, *CI*_*95*_ = {7.58, 15.54}), *F* (1, 52) = 10.152, *p* = .002, *η*^*2*^ = .163, as well as stronger decreases in positive affect (*M*_*Δpositive affect*_ = -20.08, *se* = 3.31, *CI*_*95*_ = {-26.89., -13.26}) compared to trait-passive-reactive individuals (*M*_*Δpositive affect*_ = -9.36, *se* = 1.45, *CI*_*95*_ = {-12.33, -6.39}), *F* (1, 52) = 8.78, *p* = .005, *η*^*2*^ = .145. There were no significant trait-differences in responses to the positive condition.

Trait-proactivity thus moderated reported affective responses to the negative affect manipulation, but not to the positive affect manipulation. A similar pattern occurred for the physiological reactions. In the proactive group, participants responded stronger to the negative stimuli (*M*_*magnitude*_ = 0.23, *se* = .03, *CI*_*95*_ = {0.17, 0.30}) than to the neutral (*M*_*magnitude*_ = 0.14, *se* = .03, *CI*_*95*_ = {0.08, 0.19}) and positive stimuli, (*M*_*magnitude*_ = 0.13, *se* = .02, *CI*_*95*_ = {0.09, 0.17}), *F* (1, 85) = 7.849, *p* = .006, *η*^*2*^ = .085. In the passive-reactive group, this difference was not significant, *F* (1, 75) = 2.276, *p* = .136, *η*^*2*^ = .030. This indicates that proactive people show more arousal in response to negative affective stimuli than passive-reactive ones, but do not show more arousal in response to positive affective stimuli.

**Fig 5:**
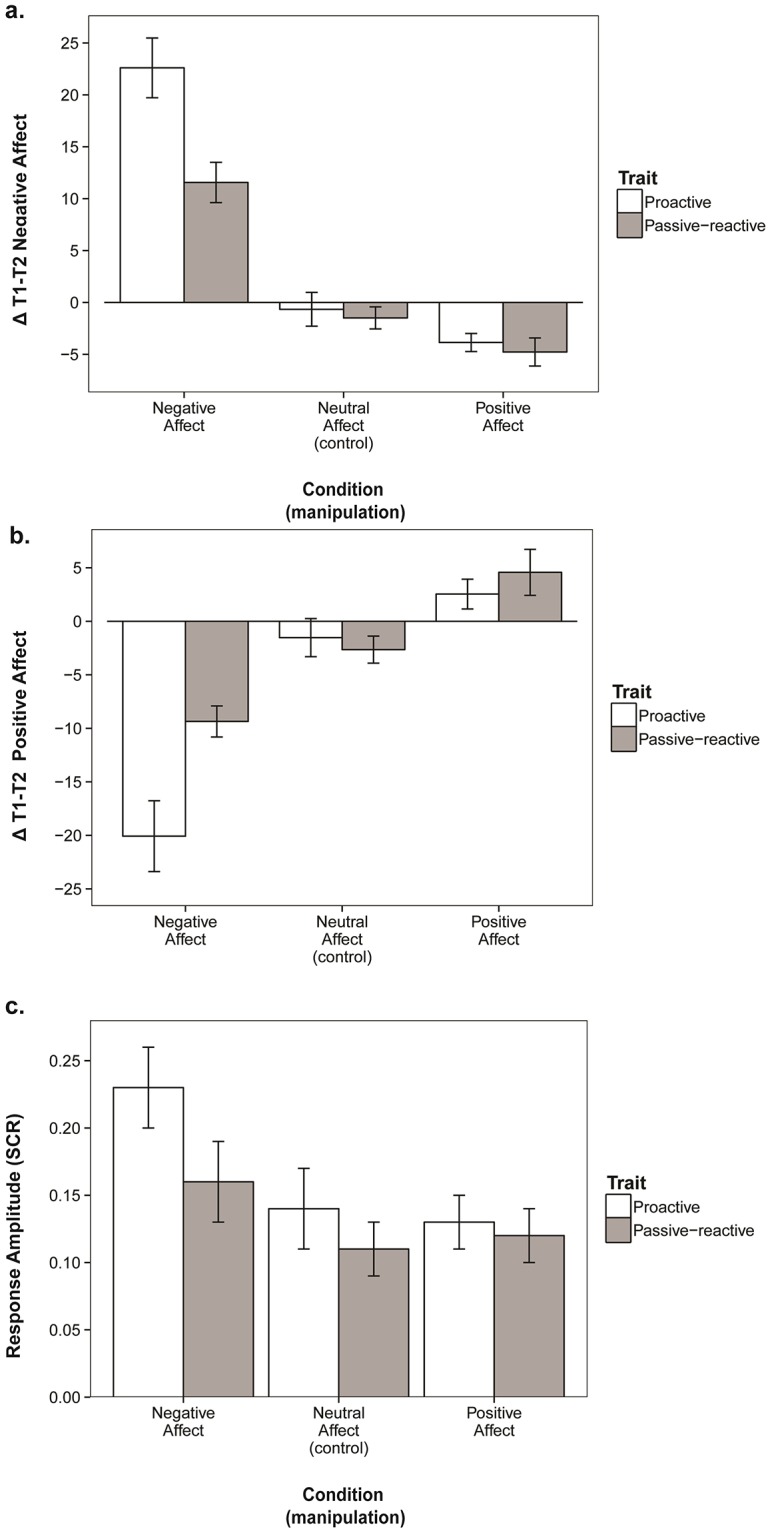
Affective responses to the affect manipulations for trait-proactive and passive-reactive participants. Dependent variables are **(a)** the increase between T1 and T2 in reported negative affect, **(b)** the decrease between T1 and T2 in reported positive affect, and **(c)** skin conductance response magnitude (SCR). Moderated by trait-proactivity (group is based on a mean-split).

An overview of the interactions between trait-proactivity and affective responses to the manipulations can be found in Table 4 and Fig 5. An overview of all hypotheses of the study can be found in Table 5.

**Table 4:** Interaction effects of affect condition * trait-proactivity on changes in affect from baseline to after the manipulation (Δ T1 –T2).

		Affect Condition					
Dependent	Moderator	Negative		Neutral (control)		Positive	
Affective Changes (Δ T1-T2)	Trait-proactivity	*M*	*se*	*M*	*se*	*M*	*se*
Negative Affect	Proactive	22.60**	2.88	-.66	1.63	-3.86	.87
	Passive-reactive	11.56	1.94	-1.49	1.06	-4.77	1.35
Positive Affect	Proactive	-20.08**	3.31	-1.53	1.78	2.54	1.39
	Passive-reactive	-9.36	1.45	-2.65	1.26	4.57	2.15
Response Magnitude (SCR)	Proactive	0.23**	.03	.14	.03	0.13	.02
	Passive-reactive	0.16	.03	.11	.02	0.12	.02

Note.

* *p* < .05,

** *p* < .01,

*** *p* < .001,

Compared to control condition. '—' reflects affective decreases, otherwise, affective increases

**Table 5:** Overview of hypotheses and their confirmation.

Confirmed	Hypothesis	Independent		Moderator		Dependent
	*H1a*	Positive affect			↑	Proactive behavior
*	*H1b*	Negative affect			↑	Proactive behavior
*	*H1c*	Affect	*	Trait-proactivity	↑	Proactive behavior
*	*H2a*	Proactive behavior			↑	Positive affect
	*H2b*	Proactive behavior			↓	Negative affect
*	*H2c*	Proactive behavior	*	Trait-proactivity	↑	Affect
*	*H3*	Affect manipulation	*	Trait-proactivity	↑	Affective responses, physiological reactions

Confirmed hypotheses are marked with an asterisks *

## Discussion

Proactive people do not wait for things to happen, they make things happen themselves. Although the importance of this trait and behavior for personal progress have been emphasized strongly, there is a lack of experimental research on what drives people to take initiative and be proactive. Research suggests that affect might play a role, but so far, evidence has been correlational. Therefore, we experimentally investigated whether positive and negative affective states drive proactive behavior, whether changes in affect also result from engaging in proactive behavior, and whether these effects differ for trait-proactive vs. passive-reactive individuals, and finally, we explored whether trait-proactive individuals are more sensitive to negative affective stimuli than passive-reactive individuals. Our results showed that trait-proactive and passive-reactive people indeed differed in 3 respects. First, compared to neutral affective states, positive affect made proactive individuals less proactive, whereas negative affect made passive-reactive individuals more proactive. Second, being proactive increased subsequent positive affect in proactive individuals, whereas it decreased subsequent negative affect in passive-reactive ones. And third, proactive individuals showed stronger self-reported and physiological reactions to negative affective stimulation than passive-reactive people.

### Theoretical implications

There are several theoretical implications of our findings regarding the differences between trait-proactive versus passive-reactive people. The first implication is cognitive-affective. Since positive affect is often linked to broad thinking whereas negative affect is linked to more systematic and narrow thinking [19, 29, 49, 50], our results indicate that systematic, narrow and persistent thought may be more important for proactivity than broad and flexible thinking. This is supported by the finding that passive-reactive individuals were most proactive in the negative condition, whereas proactive individuals were equally proactive under negative and neutral affective circumstances. As proactive people tend to be goal directed, persistent, and active in general [2], they may need less situational (affectively stimulated) systematic and persistent mental activation as this is already their default mental approach. This is also suggested by the finding that positive affect decreased proactivity in proactive individuals. Perhaps it changed their otherwise systematic, localized, and persistent attention focus to a broader, more flexible focus.

This explanation conflicts with current proactivity theories, in which positive affect and broad-flexible cognitive processing are assumed to enhance proactive behaviour [9, 10]. Our results point in two directions. First, it could be that previously found correlations between positive affect and proactivity are primarily found because positive affect results from proactive behaviour, not because it enhances it. This explanation is supported by longitudinal work that links proactive behavior to increased positive affect [15], and by our finding that the more proactive our participants behaved (and the higher their trait-proactivity), the more their positive affect increased. Alternatively, or additionally, the relationship between positive feelings and proactivity might be driven solely by the motivational pathway, such that only positive emotions that are self-efficacy enhancing (pride, enthusiasm, optimism) enhance proactive behaviour because they increase expectations that the behaviour will be effective, but more diffuse, core positive affect does not.

Our results regarding negative affect as a driver and consequence in passive-reactive individuals do fit with previous research indicating that negative affective triggers such as job-stressors can enhance proactive behavior [14, 15], but extend these findings in three ways. First, the influence of core negative affect on proactivity supports the idea that systematic-persistent thought is important for executing proactivity. Second, we investigated core negative affect unrelated to the job-context. The fact that this core negative state increased proactivity and that proactive behavior made participants feel less negative and more positive, suggests that proactivity can act as an affect-focused coping mechanism (directly regulating affect) rather than a problem-focused coping mechanism (addressing the source of the negative affect and ‘solving the problem’). Third, negative affective drivers and consequences are strongest for passive-reactive individuals, indicating that trait-proactivity needs to be included in studies on affect and proactivity.

### Alternative explanations

Although one way to look at the affect proactive behavior relationship is cognitive-affective, presumably, there are motivational mechanisms at play as well. We found several indications that proactive behavior can be (explicitly or implicitly) used to regulate affective experiences. The reasons for regulation however, as well as the type of affect (negative or positive) that is regulated, may differ between proactive and passive-reactive individuals. First, in the negative affect condition, passive-reactive individuals experienced less negative affect and activation than proactive individuals, while at the same time they showed stronger proactive behavior increases than proactive individuals. The negative affect manipulation thus strongly affected passive-reactive individuals’ proactivity, but not their affect or their arousal, whereas for proactive individuals, the manipulation strongly influenced affect and arousal, but not their proactivity. One explanation might be that passive-reactive individuals need stronger affective activation to become proactive *because* they are less sensitive to negative affective triggers than proactive individuals.

As noted, trait-proactivity is related to creativity. Genetic research implies that mentally healthy creative people share genetic variants with people with psychiatric mood-disorders [51–53]. This may also hold for proactive people. Trait-proactive people might be biologically predisposed for sensitivity to negative affective stimulation, and passive-reactive people might need more severe (negative) activation to become proactive as they are less sensitive to negative affective stimuli. At the same time, proactive people do not tend to be emotionally unstable: both proactive trait and behavior are unrelated to neuroticism [7]. Other research shows that particularly neuroticism is related to physiological responses to negative emotional stimulation [43]. So even though proactive individuals show sensitivity to negative affective stimulation like neurotic individuals do, proactive people are not emotionally unstable (i.e. neurotic). One explanation could be that unlike neurotic people, proactive people have learned to use proactive behavior to regulate their affective states. In our study, proactive participants showed steep increases in positive affect and energy after they engaged in proactive behavior. At the same time, proactive individuals showed least proactive behavior when positive affect was already induced. This indicates that perhaps, proactive individuals are motivated to be proactive to enhance future positive affect. Following an affect-motivational approach, if proactive individuals are motivated to enhance future positive affect, being in a positive state already relieves the drive to be proactive.

While proactive individuals might be motivated to enhance (or approach) future positive affective experiences, our findings suggest that perhaps, passive-reactive individuals are motivated to show persistent initiative when they want to avoid or suppress current negative affective experiences. Passive-reactive individuals might only become proactive when there is a need to reduce or avoid negative feelings (i.e., when they experience strong negative affect). Taken into account that they may be less emotionally sensitive than proactive individuals, they need stronger negative activation to feel the need to reduce negative affect. The fact that passive-reactive individuals may become proactive through a negative affect or avoidance-motivational pathway is supported by the fact that passive-reactive people where most proactive when a negative affective state was induced, and they showed decreases in negative affect after proactive behavior. This contrasts with the approach-motivational pathway (engaging in proactivity to enhance future positive affect) just described for proactive individuals, who showed decreased proactivity in a positive state, and increased positive affect after proactivity. This affect-motivational explanation fits with the general description of trait-proactivity: whereas proactive people take *anticipatory* action to change (goal to achieve or approach future positive affect), passive-reactive people *react* to situational demands (goal to reduce or avoid current negative affect).

### Limitations and future research

Our study has methodological merit: separate measures of trait-proactivity and behavior, reported affective experiences over time, a physiological measure of activation, and an experimental design. However, it also has limitations. First and foremost, we did not explicitly measure broad/flexible or narrow/persistent thinking. Future research where broad and narrow thinking is induced [29] is necessary to support the idea that it is indeed cognitive persistence underlying the effect of negative affect on proactive behaviour. Second, we proposed that proactive individuals might be more focused on approaching future positive affect, whereas passive-reactive people might try to avoid current negative affect. However, this line of reasoning needs further investigation before any conclusions can be drawn. Future research could investigate trait-proactivity in relation to affective approach and avoidance motivation [54] and time perspective [55]. The approach and avoidance motivational aspects of affect have also recently received attention in the cognition and affect literature. Recent research has indicated that there are specific positive approach oriented emotions (such as desire) that narrow the scope of attention, whereas there are also specific negative emotions (such as sadness) that broaden the scope of attention. More research on different types of affect, their effects on cognition, and behavior, is necessary to draw conclusions about the type of thinking involved in proactive behavior.

Next, our results regarding the affect regulating consequences of proactivity are limited to the short time window of our lab study. While our study shows that proactive behavior is likely to increase positive affect and decrease negative affect, a recent diary study in organizations [56] has shown that proactive behavior can also have negative consequences because it can increase stress levels. These stress levels may (a) be a remainder of a stressor that caused proactivity in the first place, (b) indicate that proactivity can drain resources (which is also supported by the fact that daily proactivity was related to bed-time fatigue) [56], or (c) result from ineffectiveness of proactive behavior. In organizational settings, proactive behavior is not always successful, and may, if unsuccessful, result in relational conflict and stress. Connecting this explanation to our finding that trait proactive individuals might be more sensitive to negative affective stimulation, trait proactive individuals might be particularly vulnerable to experience strain or stress if proactive behavior is unsuccessful. Further research on how trait proactivity and more prolonged successful and unsuccessful proactive behavior may interact to influence affect, stress, and burn-out, is a very interesting avenue for future research.

We also need to be careful about treating reported affect and physiological activation levels as two measures of the same variable [57]. Even though we do not claim they are, our results indicate that affect, physiological activation levels, and proactivity are intertwined. We need further research to disentangle whether we can separate affective valence and mere activation in relation to trait-proactivity and behavior. It may also be that affective sensitivity of trait-proactive individuals is merely activation based, and not so much affect-based, or that proactive people are also more sensitive to positive emotional stimulation. Trait-proactivity is related to extraversion [7], and extraversion is related to increased amygdala responses to positive emotional stimulation [58]. Here we reasoned and found that proactive people would be more sensitive to negative affective stimuli, but it might thus also be that proactive people are generally more emotionally sensitive. We did not find an effect for positive affect, but it may be that our positive affect manipulation was not strong enough to detect this. On the other hand, the positive affect manipulation was strong enough to decrease proactivity in proactive people, showing it was strong enough to influence behavior.

Another point of note is that we manipulated core positive affect in our experiment; results for more specifically confidence-related positive emotions such as optimism, pride, and enthusiasm are likely to differ from the results of core affect as different causal mechanisms then come into play (e.g., increased self-efficacy affecting proactivity). These confidence-related emotions may thus stimulate proactive behavior as currently theorized in the proactivity literature [9, 11], but our results suggest that this might be primarily via other pathways than broad and flexible thinking, such as through enhancing efficacy and outcome expectancies of proactive behavior. However, future research using other ways (besides affect) to influence or measure cognitive pathways are needed to further unravel which mental processes foster the execution of proactive behavior.

Last, our design did not allow for an investigation of the possible interaction between positive and negative affect, or perhaps the benefits of alternating positive and negative affect or positive and negative emotions. Proactive behaviour is a complex behaviour that consists of several stages. There are three general theoretical models [1, 2, 11] of the proactive process that follow similar sequences of thought / action patterns. These models suggest that the proactive process starts with (1) the generation of a proactive goal, followed by (2) a plan for action, (3) enactment of the plan while persisting proactive behavior over time, and (4) reflection upon success or failure of proactivity. One question that may be interesting to study, is how positive and negative affect and broad versus narrow thinking help or hinder these phases, and whether they can trigger the transitions from one phase to another. For example, broad thinking might be useful in the generation phase because flexible broad thought and positive affect have often been linked to creativity, whereas persistence might be more useful in the planning or enactment phase [59]. Since our experiment focused primarily on planning and enactment, this could be why we found that negative affect was most proactivity enhancing. Future studies where other phases of proactivity are also involved, might find different effects.

### Contributions and conclusion

This study integrates the organizational proactivity and the affect- and emotion-regulation literature. We add to a broader stream of research on the functionality of negative affect indicating that negative affect influence the quality of decisions and behaviour through cognition and motivation [60]. We introduce proactivity as a change and control oriented behavior with possible affect-regulating properties. We expand current theories of proactivity by showing that core positive affect may be a result of proactivity rather than an antecedent, whereas core negative affect might be a driver of proactivity that is new to the literature and thus needs further attention. We add nuance to current work on proactivity by differentiating between core affect, and more specific, goal directed emotions (e.g., hopelessness, pride, enthusiasm), thereby enhancing knowledge about possible cognitive mechanisms underlying the relationship between affect and proactivity. While the literature assumes that positive affect and broad and flexible thinking increase proactivity, this first experimental study shows more evidence for a negative affect pathway through systematic and persistent thought. Last, we found some indications that proactive and passive-reactive individuals are affectively different from one another. As a consequence, they may have different affective reasons for proactive behavior. Whereas proactive individuals may show proactive behavior because they want to feel better in the future, passive-reactive individuals may only show proactive behavior when they feel bad now.

## Supporting information

S1 Appendix Supplementary materials do you feel like being proactive today. (DOCX)
