# Supplementary material for: Do you feel like being proactive today? Trait-proactivity moderates affective causes and consequences of proactive behavior
Source: PLoS One. 2019 Aug 13;14(8):e0220172. doi: 10.1371/journal.pone.0220172 (PMC6692000; doi:10.1371/journal.pone.0220172)
Supplement: S1 Appendix — (DOCX) [file pone.0220172.s001.docx]

Supplementary materials do you feel like being proactive today

**Selection of Musical Material**

For auditory stimulation, we selected John Adam’s ‘Common tones in simple time’ from Västfjäll (2001) for the neutral condition. As most of the excerpts from Västfjälls’ review enhance very specific emotions such as only sadness (slow music) or fear (fast music), we created a panel of 4 music experts (including the first author) to select more generally positive and negative activating excerpts. These excerpts where discussed with 5 naïve students without musical knowledge. We unanimously chose a segment of Clint Mansell’s movie soundtrack ‘Requiem for a dream’ for the Negative condition, as all panelists felt it evoked a cocktail of negative feelings but was activating nonetheless. In the Positive condition, we used a compilation of Shostakovich’s 1st and 2nd ballet and jazz suites that were selected for their upbeat, happy and elated sound that fitted best with the selected pictures. The music excerpts were presented through headphones for the same duration as the picture presentation.

**Proactive Behavior Measure Validity**

Since this is the first attempt to capture proactive behavior in the lab, our first step was to validate the task by comparing it to our trait-proactivity construct. Here, we report the overall correlations between proactive measures (all affect conditions), as well as the correlations in the control condition separately. Overall, proactive behavior was positively related to the pre-test of trait-proactivity, *r* (162) = .233, *p* =.003. In the control condition, the relationship was stronger, than in the total sample, *r* (50) = .456, *p* =.001. This indicates that our behavioral measure is strongly related a well validated multi-source trait-proactivity scale, especially under neutral situational circumstances. We thus assume that we captured important aspects of proactivity in the lab.

**Affect Factor analysis**

We performed separate exploratory factor analyses (PCA, Varimax rotation) on the 10-item valence part of affect and on the 10 item physical activation part of the questionnaire for all 3 time-points to see whether we would find stable negative vs. positive factors for both valence and activation across time. All rotations converged within 3 iterations, with two stable factors reflecting positive and negative valence (KMO range .907 - .931) and two relatively stable factors reflecting negative tension and positive energy in the physical activation part (KMO range .741 - .833). All items, factor loadings and reliabilities can be found in tables S1a and b. For scholars who are interested in focusing specifically on activation or valence, Table S3 reflects correlations between these separate factors and the proactivity constructs.

**Table A. Factor Loadings for affective valence**

| Time | 1 | |  | 2 | |  | 3 | | |
| --- | --- | --- | --- | --- | --- | --- | --- | --- | --- |
| Factor | Positive | Negative |  | Positive | Negative |  | Positive | Negative | |
| Explained variance | 59% | 14% |  | 72% | 10% |  | 61% | 16% | |
| Items |  |  |  |  |  |  |  |  | |
| Pleasant | **.793** | -.283 |  | **.791** | -.474 |  | **.833** | -.386 | |
| Good | **.856** | -.285 |  | **.812** | -.478 |  | **.850** | -.379 | |
| Happy | **.785** | -.377 |  | **.834** | -.453 |  | **.866** | -.253 | |
| Good mood | **.703** | -.441 |  | **.830** | -.431 |  | **.826** | -.324 | |
| Cheerful | **.870** | -.085 |  | **.902** | -.167 |  | **.900** | -.086 | |
| Bleak | -.427 | **.671** |  | -.464 | **.772** |  | -.324 | **.817** | |
| Anxious | -.071 | **.801** |  | -.220 | **.856** |  | -.073 | **.858** | |
| Sad | -.203 | **.823** |  | -.293 | **.813** |  | -.250 | **.809** | |
| Reluctant | -.355 | **.590** |  | -.383 | **.737** |  | -.287 | **.710** | |
| Bad | -.485 | **.705** |  | -.465 | **.772** |  | -.416 | **.765** | |
| © counterbalanced items  Factor loading > .300 are printed in **bold** | | | | | | | | |  |

**Table B. Factor Loadings for physical activation**

| Time | 1 | |  | 2 | |  | 3 | |
| --- | --- | --- | --- | --- | --- | --- | --- | --- |
| Factor | Energy | Tension |  | Energy | Tension |  | Energy | Tension |
| Explained variance | 38% | 16% |  | 19% | 44% |  | 18% | 36% |
| Items |  |  |  |  |  |  |  |  |
| Lively | **.811** | .017 |  | **.847** | -.175 |  | **.834** | -.001 |
| Apathetic © | **.763** | .200 |  | **.752** | .335 |  | **.651** | .305 |
| Energized | **.856** | -.016 |  | **.880** | -.171 |  | **.880** | .040 |
| Tired © | **.737** | .141 |  | **.785** | .019 |  | **.593** | .144 |
| Emotionally affected | .333 | **.601** |  | .145 | **.795** |  | -.271 | **.735** |
| Tensed | .112 | **.731** |  | .231 | **.755** |  | -.272 | **.654** |
| Increased heartbeat | -.136 | **.819** |  | -.109 | **.806** |  | -.007 | **.713** |
| Cold | .359 | .288 |  | .250 | **.613** |  | -.041 | **.689** |
| Goosebumps | .387 | .151 |  | .192 | **.684** |  | -.067 | **.692** |
| Relaxed © | .231 | **.743** |  | .460 | **.575** |  | .542 | **.406** |
| © counterbalanced items  Factor loading > .300 are printed in **bold** | | | | | | | | |

**Table C. All interaction effects involving Trait-Proactivity**

|  |  |  |  | **Trait-Proactivity** | | |
| --- | --- | --- | --- | --- | --- | --- |
|  |  |  |  | **Mean-split** | **Continuous** | |
| ***Q 1*** | Dependent | Proactive Behavior | *F* | 4.40* | 3.13* | |
|  | Interaction | Affect Condition * Trait-Proactivity | *p* | .014 | .047 | |
|  |  |  | *η²* | .053 | .049 | |
| ***Q2a*** | Dependent | Positive affect increases during proactive behavior | *F* | 6.55* | 5.48* | |
|  | Interaction | Proactive Behavior * Trait-Proactivity | *p* | .011 | .021 | |
|  | Controls | Affect condition, Time | *η²* | .041 | .034 | |
| ***Q2b*** | Dependent | Negative affect decreases during proactive behavior | *F* | 2.17 | 5.01* | |
|  | Interaction | Proactive Behavior * Trait-Proactivity | *p* | .140 | .027 | |
|  | Controls | Affect condition, Time | *η²* | .013 | .032 | |
| ***EQ*** | Dependent | Negative Affect Increases during manipulation | *F* | 5.72** | 4.31* |  |
|  | Interaction | Affect Condition * Trait-Proactivity | *p* | .004 | .015 |  |
|  |  |  | *η²* | .068 | .052 |  |

**Note.** *p* < .05, ** *p* < .01, *** *p* < .001.

**Table D. Correlations between all affect factors and proactive constructs**

|  |  |  | **Proactive Constructs** | |
| --- | --- | --- | --- | --- |
|  |  |  | **Behavior** | **Trait** |
| **Affect after manipulation  (Δ T1 and T2)** | Negative | Total | **.159*** | .**157*** |
|  |  | Valence | .135 | .141 |
|  |  | Tension | **.165^*^** | .145 |
|  | Positive | Total | -.106 | -.146 |
|  |  | Valence | -.081 | **-.168^*^** |
|  |  | Energy | -.117 | -.080 |
| **Affect after proactive behavior  (Δ T2 and T3)** | Negative | Total | -.153 | -.106 |
|  |  | Valence | **-.187^*^** | -.097 |
|  |  | Tension | -.080 | -.090 |
|  | Positive | Total | **.298***** | .109 |
|  |  | Valence | **.224^**^** | .105 |
|  |  | Energy | **.334^**^** | .090 |

**Note.** *p* < .05, ** *p* < .01, *** *p* < .001.
